# The construction and validation of an RNA binding protein-related prognostic model for bladder cancer

**DOI:** 10.1186/s12885-021-07930-5

**Published:** 2021-03-08

**Authors:** Fengxia Chen, Qingqing Wang, Yunfeng Zhou

**Affiliations:** 1 Hubei Cancer Clinical Study Center, Hubei Key Laboratory of Tumor Biological Behaviors, Zhongnan Hospital, Wuhan University, Wuhan, China; 2 Department of Radiation Oncology and Medical Oncology, Zhongnan Hospital, Wuhan University, Wuhan, China

**Keywords:** Bladder cancer/BLCA, TCGA, RNA binding proteins, Prognostic model, Survival

## Abstract

**Background:**

RNA-binding proteins (RBPs) play crucial and multifaceted roles in post-transcriptional regulation. While RBPs dysregulation is involved in tumorigenesis and progression, little is known about the role of RBPs in bladder cancer (BLCA) prognosis. This study aimed to establish a prognostic model based on the prognosis-related RBPs to predict the survival of BLCA patients.

**Methods:**

We downloaded BLCA RNA sequence data from The Cancer Genome Atlas (TCGA) database and identified RBPs differentially expressed between tumour and normal tissues. Then, functional enrichment analysis of these differentially expressed RBPs was conducted. Independent prognosis-associated RBPs were identified by univariable and multivariable Cox regression analyses to construct a risk score model. Subsequently, Kaplan–Meier and receiver operating characteristic curves were plotted to assess the performance of this prognostic model. Finally, a nomogram was established followed by the validation of its prognostic value and expression of the hub RBPs.

**Results:**

The 385 differentially expressed RBPs were identified included 218 and 167 upregulated and downregulated RBPs, respectively. The eight independent prognosis-associated RBPs (*EFTUD2*, *GEMIN7*, *OAS1*, *APOBEC3H*, *TRIM71*, *DARS2*, *YTHDC1*, and *RBMS3*) were then used to construct a prognostic prediction model. An in-depth analysis showed lower overall survival (OS) in patients in the high-risk subgroup compared to that in patients in the low-risk subgroup according to the prognostic model. The area under the curve of the time-dependent receiver operator characteristic (ROC) curve were 0.795 and 0.669 for the TCGA training and test datasets, respectively, showing a moderate predictive discrimination of the prognostic model. A nomogram was established, which showed a favourable predictive value for the prognosis of BLCA.

**Conclusions:**

We developed and validated the performance of a prognostic model for BLCA that might facilitate the development of new biomarkers for the prognostic assessment of BLCA patients.

## Background

Bladder cancer (BLCA) is the most common malignancy in the urinary system, ranking 4th among men and 18th among women [1]. The 5-year survival rates have remained generally flat since the 1990s due to late diagnosis and limited therapeutics. Patients with ‘non-muscle-invasive’ tumours are easier to treat and have lower mortality rate compared to those in patients with tumours that have grown into the muscle wall or beyond [2]. Currently, BLCA screening heavily relies on cystoscopy, upper urography, urine cytology, and computed tomography (CT) [3]. Cystoscopy is an invasive examination method that is also expensive and uncomfortable for patients. However, urine cytology is less sensitive. The measurement of circulating biomarkers is a promising diagnostic method owing to their relative availability in serum and plasma [2]. Thus, there is an urgent need to identify early diagnostic biomarkers and prognostic indexes to improve the treatment effects and survival rate of BLCA.

RNA-binding proteins (RBPs) interact with RNA to form ribonucleoprotein complexes that regulate RNA expression and function [4]. As important participants in post-transcriptional regulation, RBPs are involved in almost all post-transcriptional regulation processes, including RNA splicing, translation, transport, localisation, degradation, and stabilisation [5]. RBP dysregulation has been reported in multiple cancers, which affects tumorigenesis and development [5]. However, the knowledge of RBP-related mechanisms in the development of cancer remains rudimentary and inconclusive. Therefore, clarification of the roles of RBPs in BLCA will help us to better understand tumour pathogenesis and develop prognostic and response biomarkers.

Recently, various RBP-related mechanisms in cancer onset and progression have been clarified, including genomic alterations, transcriptional and post-transcriptional control, and posttranslational modifications [5]. In addition, RBPs directly or indirectly affected oncogenic and tumour-suppressive signalling pathways [6]. However, only a few RBPs have been completely studied and identified as vital players in human cancers. For example, PNO1, a novel RBP isolated from the human kidney, functions as an oncogene in urinary bladder cancer by promoting proliferation and inhibiting apoptosis of urinary bladder cancer cells [7]. ZFP36L1, a tandem zinc-finger RBP that mediates mRNA decay, acts as a tumour suppressor to regulate mRNAs involved in hypoxia and the cell cycle [8]. A recent study demonstrated that IMP3, a member of the insulin-like growth factor II messenger RNA binding protein (IMP) family, was significantly upregulated in muscle-invasive BLCA compared to non-muscular invasive tissues and could serve as an independent prognosis predictor for BLCA patients [9]. Previously, most research mainly focused on the correlation between a single or a limited number of RBPs and BLCA. A comprehensive study of RBPs functions will help us to fully understand their roles in BLCA. Therefore, this study downloaded RNA sequence data and corresponding clinical information concerning BLCA from The Cancer Genome Atlas (TCGA) database to screen for RBPs differentially expressed between tumour and normal samples. Subsequently, a series of bioinformatics analysis methods were performed based on these differential RBPs to finally identify eight independent prognosis-associated RBPs, which were then used to construct prognostic and nomogram survival models. The results of this study might facilitate the development of prognostic assessment models based on RBPs in patients with BLCA.

## Methods

### Data processing

We downloaded RNA sequence and corresponding clinical data from the TCGA database (TCGA, https://portal.gdc.cancer.gov/), including 19 normal samples and 414 BLCA samples. The negative binomial distribution method was used to identify differentially expressed RBPs between normal and BLCA samples [10]. The *limma* package (http://www.bioconductor.org/packages/release/bioc/html/limma.html) was used for analysis. Differentially expressed RBPs were screened using the criteria of false discovery rate (FDR) < 0.05 and |log2 fold-change (FC)| > 1. The R package *pheatmap* (https://cran.r-project.org/web/packages/pheatmap/index.html) was used to perform bidirectional hierarchical clustering of the expression values of the differentially expressed RBPs.

### Gene ontology (GO) enrichment and Kyoto Encyclopaedia of genes and genomes (KEGG) pathway analyses

The biological functions of the differentially expressed RBPs were systematically examined by GO enrichment and KEGG pathway analyses using the R packages *DOSE*, *clusterProfiler*, *enrichplot*, *ggplot2*, etc. Both *P* and FDR values < 0.05 were considered statistically significant.

### Protein-protein interaction (PPI) network construction and module screening

Differently expressed RBPs were submitted to the STRING database (http://www.string-db.org/) to detect PPIs [11]. The PPI network was then constructed and visualized using Cytoscape 3.7.0. The Molecular Complex Detection (MCODE) plug-in was used to screen the key modules from the PPI network with both MCODE scores and node counts > 5 [12]. *P* < 0.05 was considered statistically significant.

### Prognostic model construction

Eight independent prognosis-associated RBPs were identified by univariate and multivariate Cox regression. Afterward, the risk score model was constructed based on the expression levels and coefficients of the eight hub RBPs. The risk score of each BLCA patient was calculated using the following formula: *Risk score* = *β*1*Exp1 + *β*2*Exp2 + *β*i*Expi, where β represents the coefficient value of the independent prognosis-associated RBP, *Exp* represents the expression level of the independent prognosis-associated RBP, and *i* represents i^th^ hub RBP.

### Validating the performance of the prognostic model

The BLCA patients were divided into low- and high-risk groups according to the median risk score. Survival differences between the two groups were evaluated by the Kaplan–Meier method using log-rank tests. In addition, receiver operating characteristic (ROC) curves were used to determine the accuracy of the prognostic model [13]. Subsequently, calibration curves and the concordance index (C-index) were calculated using the rms (https://cran.r-project.org/web/packages/rms/index.html) and the survcomp (http://www.bioconductor.org/packages/release/bioc/html/survcomp.html) packages in R, respectively. A nomogram survival model was performed using the R package *rms* based on the eight independent prognosis-associated RBPs to predict the survival rate of BLCA patients at 1, 2, and 3 years. Univariable and multivariable Cox regression analyses were performed to assess the independent clinical prognostic factors in BLCA patients from TCGA.

### Verification of the prognostic value and expression levels of the hub RBPs

The prognostic value of the eight RBPs in BLCA was assessed by plotting the Kaplan–Meier survival curves using log-rank tests. The Human Protein Atlas (HPA) online database (http://www.proteinatlas.org/) was used to investigate the differential expression of the eight hub RBPs at the protein level between tumour and normal tissues.

## Results

### Screening of differentially expressed RBPs

**Fig. 1:**
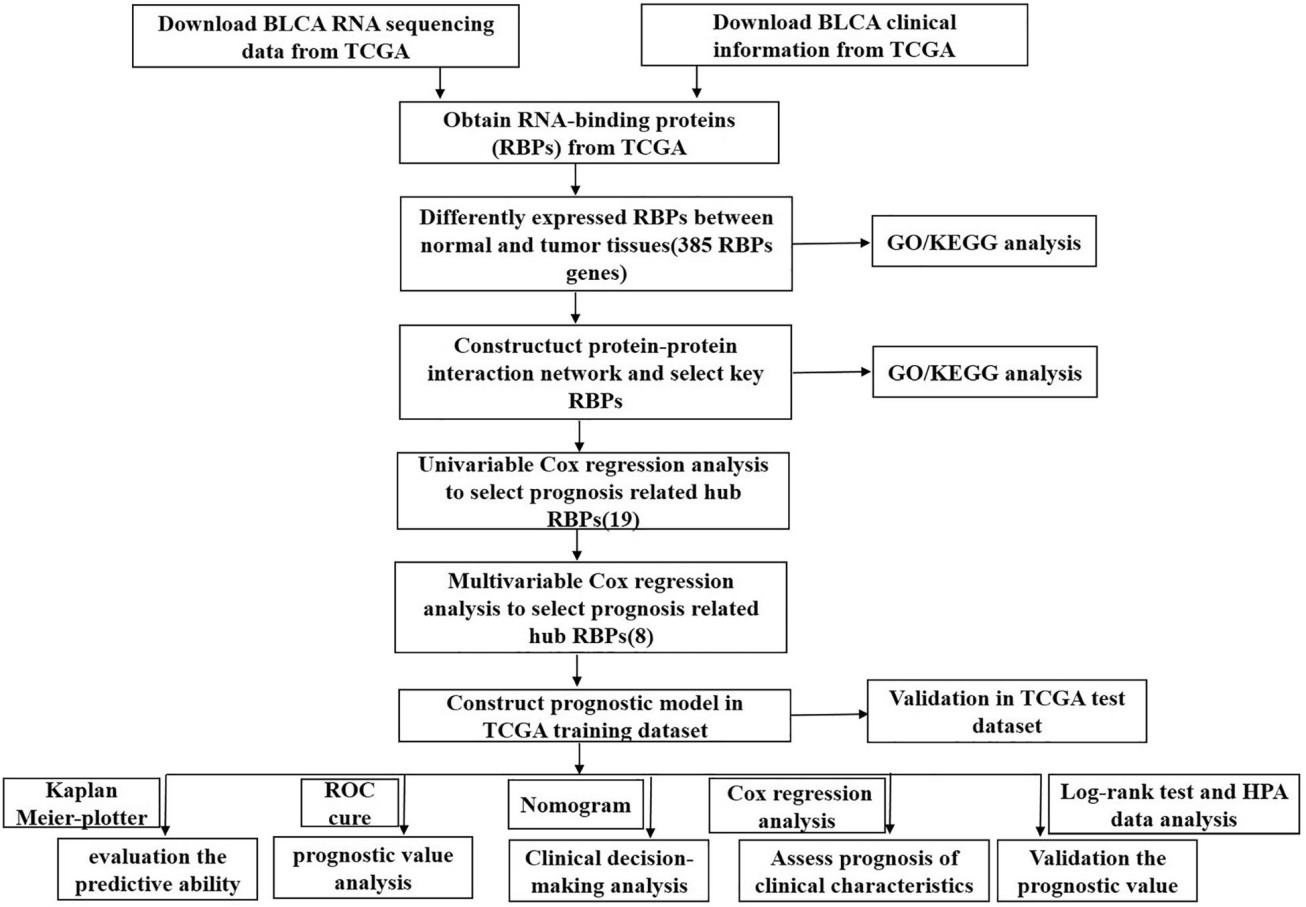
Flowchart for analysing RBPs in BLCA

**Fig. 2:**
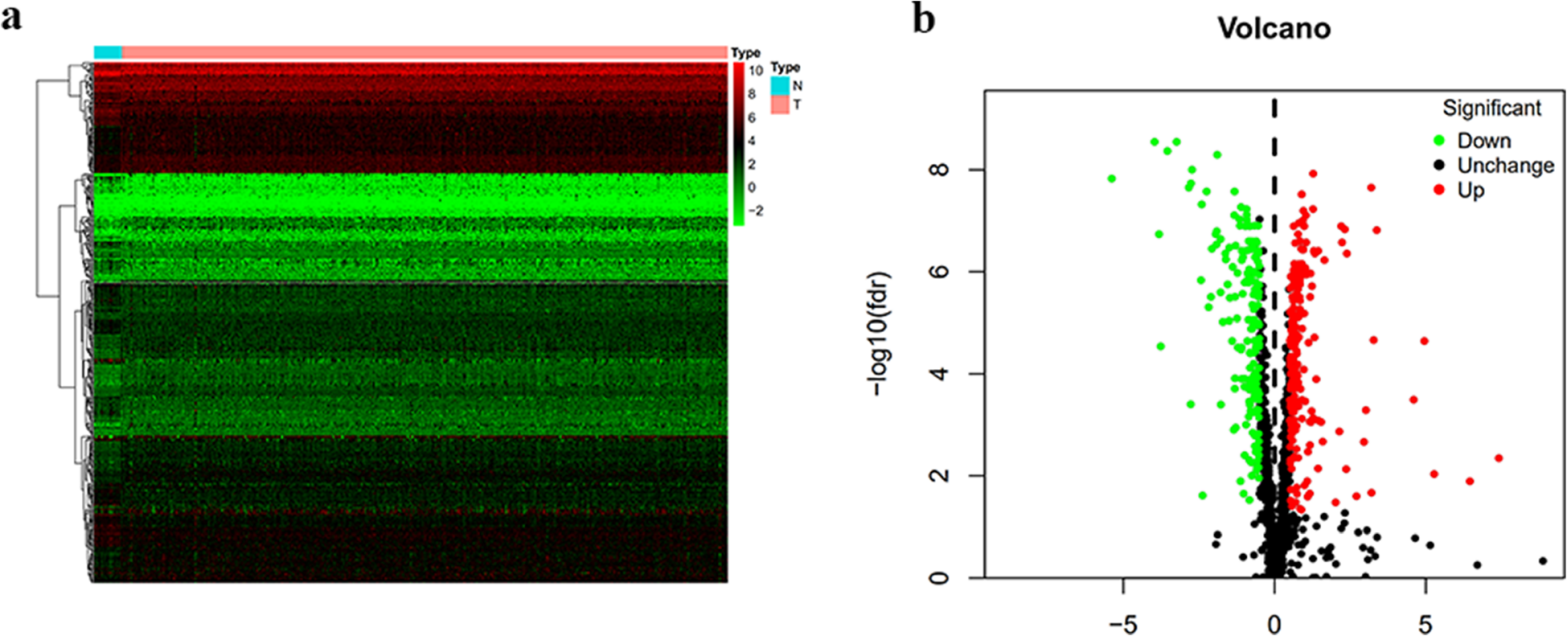
Differentially expressed RBPs between BLCA and normal tissues. **a** Heatmap of differentially expressed RBPs. **b** Volcano map of the 1542 RBPs. The red, green, and black dots indicate high, low, and no difference in expression between bladder cancer and normal tissues

This study performed a series of bioinformatics techniques to comprehensively analyse the roles and prognostic value of RBPs in BLCA. The flowchart of this study is shown in Fig. 1. We obtained RNA sequencing data and clinical information from the TCGA database containing 414 BLCA tissues and 19 normal tissues. The expression values of 1542 RBPs [4] were analysed in this study. A total of 385 differentially expressed RBPs were identified using the DEseq package that met the criteria of *P* < 0.05 and |log2 FC)| > 1.0, including 218 up-regulated and 167 down-regulated RBPs. The clustering heatmap and volcano plot of these differentially expressed RBPs are shown in Fig. 2**.**

### GO and KEGG pathway enrichment analysis of the differentially expressed RBPs

**Fig. 3:**
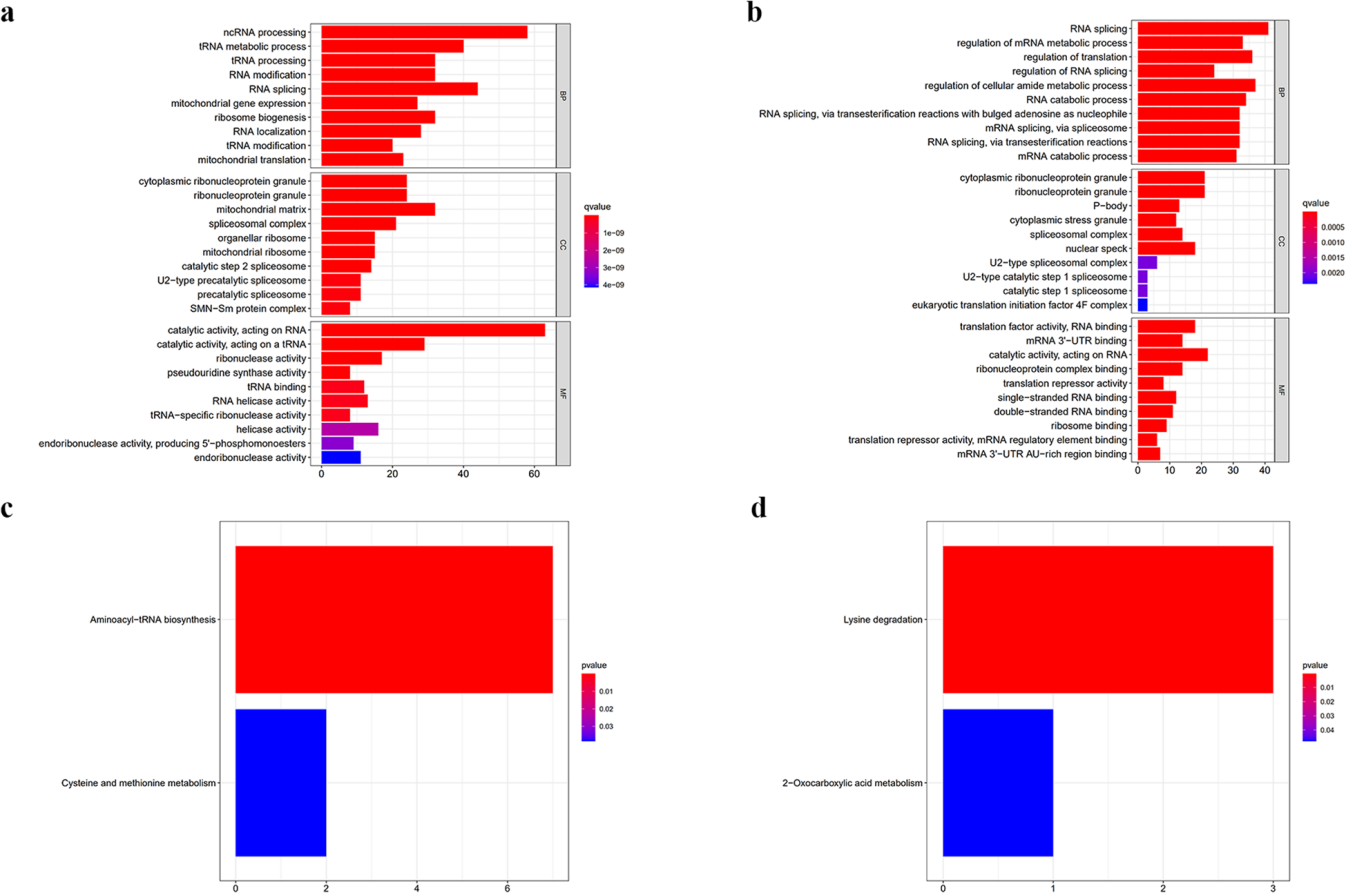
GO and KEGG pathway enrichment analysis of the differentially expressed RBPs. **a** GO enrichment analysis of upregulated expressed RBPs. **b** GO enrichment analysis of downregulated expressed RBPs. **c** KEGG pathway analysis of upregulated expressed RBPs. **d** KEGG pathway analysis of downregulated expressed RBPs

To investigate the potential function and mechanisms of the identified RBPs, we divided these differentially expressed RBPs into upregulated and downregulated groups and performed GO and KEGG pathway enrichment analyses. The GO enrichment analysis showed that the biological processes of the upregulated RBPs were mainly enriched in ncRNA processing, tRNA metabolic processes, and RNA splicing, while the downregulated RBPs were mainly enriched for RNA splicing, regulation of cellular amide metabolic processes, and regulation of translation. The cellular component analysis indicated that the upregulated and downregulated RBPs were all primarily enriched in cytoplasmic ribonucleoprotein and ribonucleoprotein granules. The molecular function analysis showed that the upregulated RBPs largely enriched in catalytic activity, acting on RNA and ribonuclease activity; meanwhile, the downregulated RBPs were mainly enriched for translation factor activity, RNA binding, and mRNA 3′-UTR binding (Fig. 3a and b).

The KEGG pathway enrichment analysis showed that the upregulated RBPs were significantly enriched in aminoacyl-tRNA biosynthesis and cysteine and methionine metabolism, while the downregulated RBPs were enriched in lysine degradation and 2-oxocarboxylic acid metabolism (Fig. 3c and d**)**.

### PPI network construction and key module screening

**Fig. 4:**
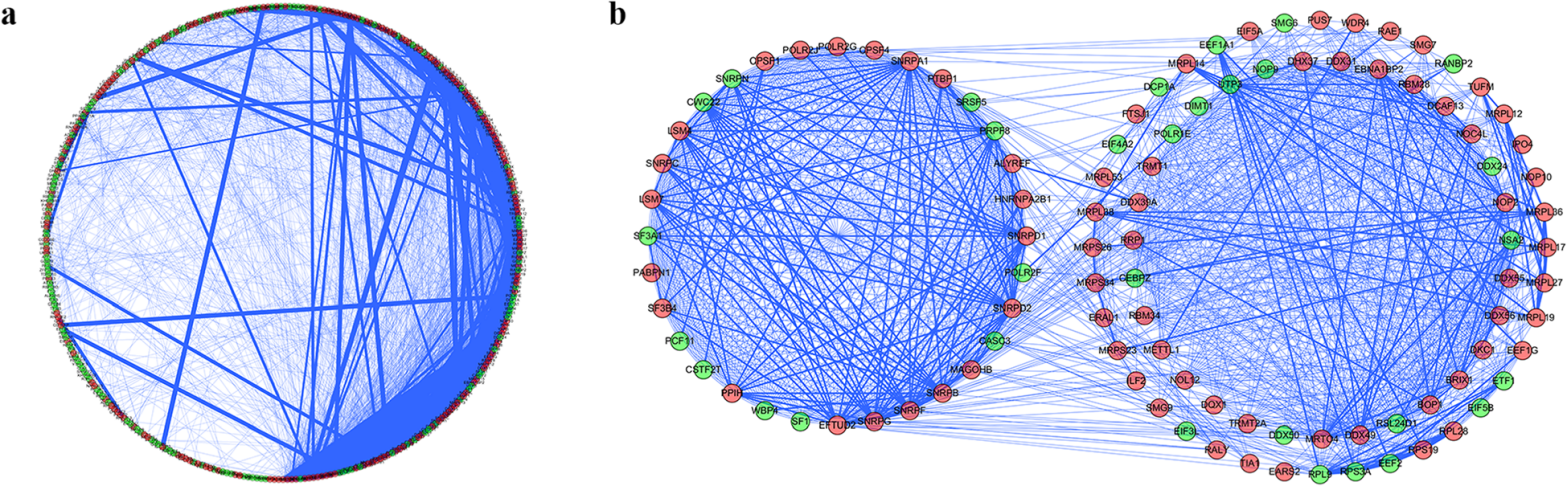
PPI network and key modules analysis. **a** PPI network of differentially expressed RBPs. **b** Key modules from the PPI network. Green circles: down-regulated RBPs. Red circles: up-regulated RBPs. The bold lines represent the top 10 interacting proteins among these expressed RBPS

To further explore the roles of differential RBPs in BLCA, Cytoscape was used to establish a PPI network comprising 373 nodes and 4063 edges based on the STRING database (Fig. 4a). Furthermore, the lines between the top 10 interacting proteins bolded according to the interaction scores. Subsequently, we used the MODE tool to analyse the co-expression network to identify the potential key modules. The most important modules comprised 104 nodes and 1151 edges (Fig. 4b). KEGG pathway analysis showed that the RBPs in these key modules were enriched for ribosome biogenesis in eukaryotes, spliceosomes, mRNA surveillance pathways, RNA polymerases, Huntington disease, cytosolic DNA-sensing pathways, RNA transport, RNA degradation, ribosomes, and legionellosis.

### Identification of prognosis-related RBPs

**Fig. 5:**
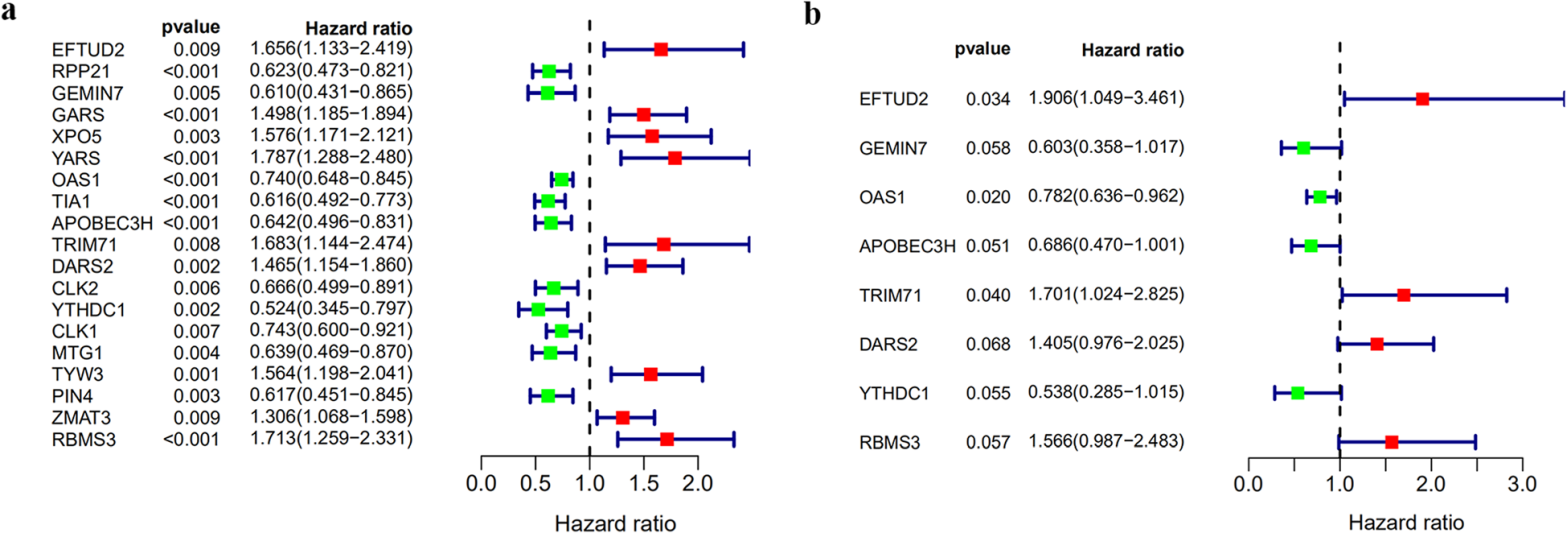
Prognosis-related RBP selection by univariable and multivariable Cox regression analyses. **a** Univariable Cox regression analysis for the identification of prognosis-associated RBPs. **b** Multivariable Cox regression analysis for the identification of independent prognosis-related RBPs

A total of 373 key differential RBPs were screened from the PPI network. To determine the association between RBPs and BLCA patients’ outcomes, univariable Cox regression analysis was conducted to evaluate the prognostic value of these key differential RBPs which identified 19 hub RBPs (Fig. 5a). Subsequently, multivariable Cox regression analysis was performed to further analyse these 19 RPBs which showed eight hub RBPs to be independent prognostic predictors in BLCA patients (Fig. 5b).

### Validation of the prognostic value and expression of hub RBPs

**Fig. 6:**
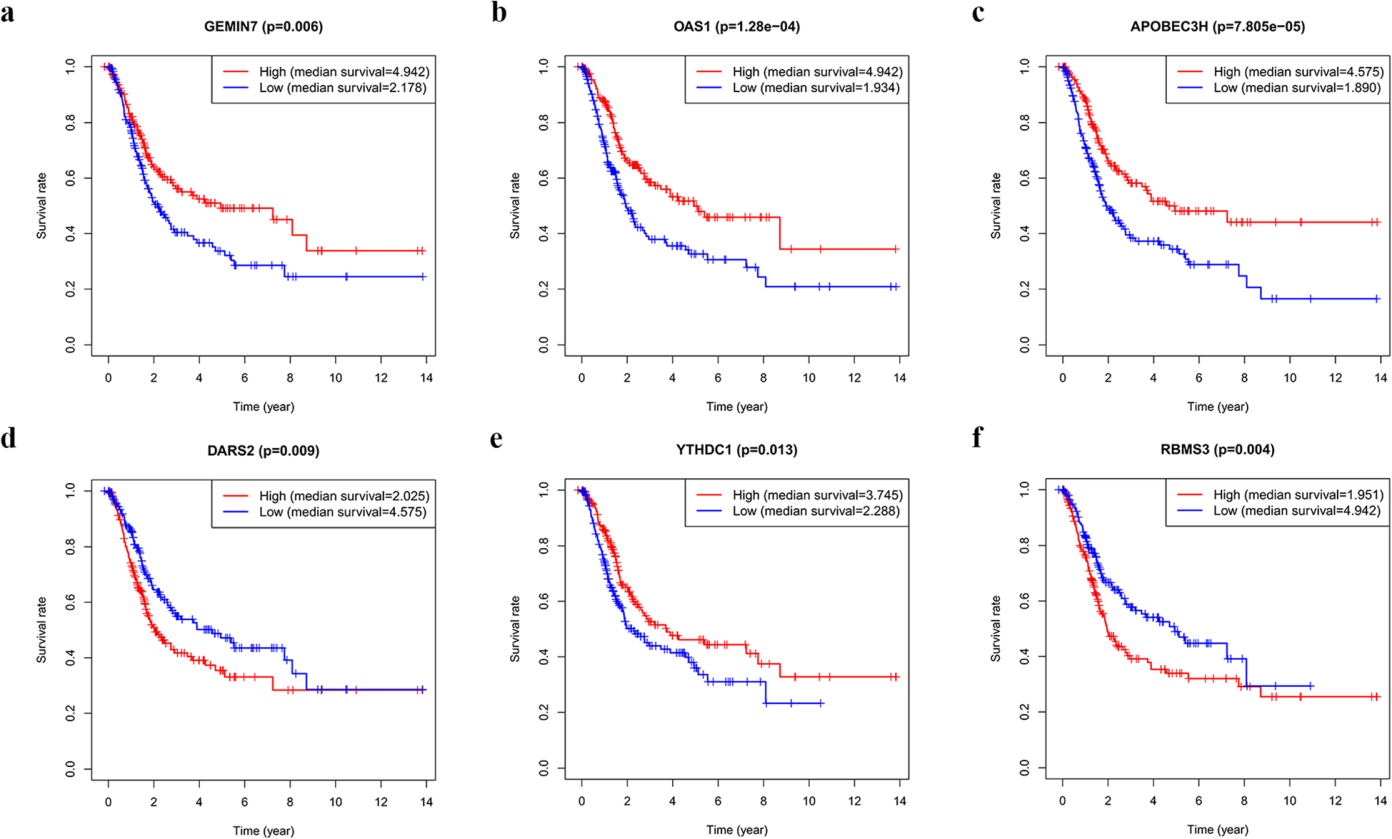
Survival analysis to verify the prognostic value of the hub RBPs in BLCA. Kaplan–Meier survival curves of **a** GEMIN7, **b** OAS1, **c** APOBEC3H, **d** DARS2, **e** YTHDC1, and **f** RBMS3. *P* < 0.05 indicated statistical significance

**Fig. 7:**
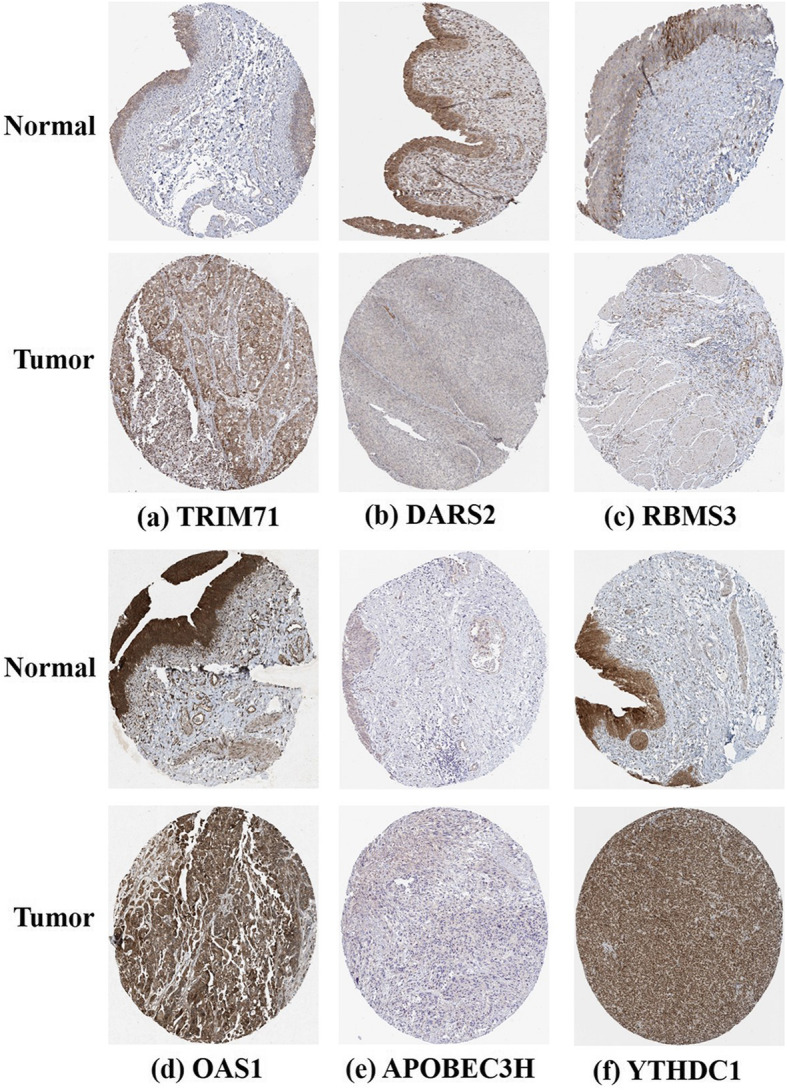
Validation of hub RBP expression in BLCA and normal bladder tissue from the HPA database. Immunohistochemistry results of **a** TRIM71, **b** DARS2, **c** RBMS3, **d** OAS1, **e** APOBEC3H, and **f** YTHDC1

To further investigate the prognostic value of these eight hub RBPs in BLCA, we plotted their Kaplan–Meier survival curves to assess their relationships with overall survival (OS). Six hub RBPs (gem nuclear organelle associated protein 7 [*GEMIN7*], 2′-5′-oligoadenylate synthetase 1 [*OAS1*], apolipoprotein B mRNA editing enzyme catalytic subunit 3H [*APOBEC3H*], aspartyl-tRNA synthetase 2, mitochondrial [*DARS2*], YTH domain containing 1 [*YTHDC1*], and RNA-binding motif, single-stranded-interacting protein 3 [*RBMS3*]) were correlated with OS in BLCA patients (Fig. 6). Furthermore, we used immunohistochemistry results from the HPA database to further explore the protein expression levels of these hub RBPs in BLCA. The results showed higher tripartite motif containing 71 [TRIM71] expression in BLCA tissues compared to that in non-tumour tissues, while DARS2 and RBMS3 expression levels were downregulated in tumour tissues. Meanwhile, there was no significant difference in the expression levels of OAS1, APOBEC3H, and YTHDC1 between tumour and normal tissues (Fig. 7). No data were available for elongation factor Tu GTP binding domain containing 2 [EFTUD2] and GEMIN7 in the HPA database.

### Construction and analysis of a prognosis-related risk score model

We established a prognosis-related risk score model based on the eight independent prognosis-associated RBPs. The risk score of each BLCA patient was calculated according to the following formula: *Risk score* = (0.6449 * ExpEFTUD2) + (− 0.5050 * ExpGEMIN7) + (− 0.2456 * ExpOAS1) + (− 0.3768 * ExpAPOBEC3H) + (0.5310 * ExpTRIM71) + (0.3403 * ExpDARS2) + (− 0.6204 * ExpYTHDC1) + (0.4484 * ExpRBMS3).

**Fig. 8:**
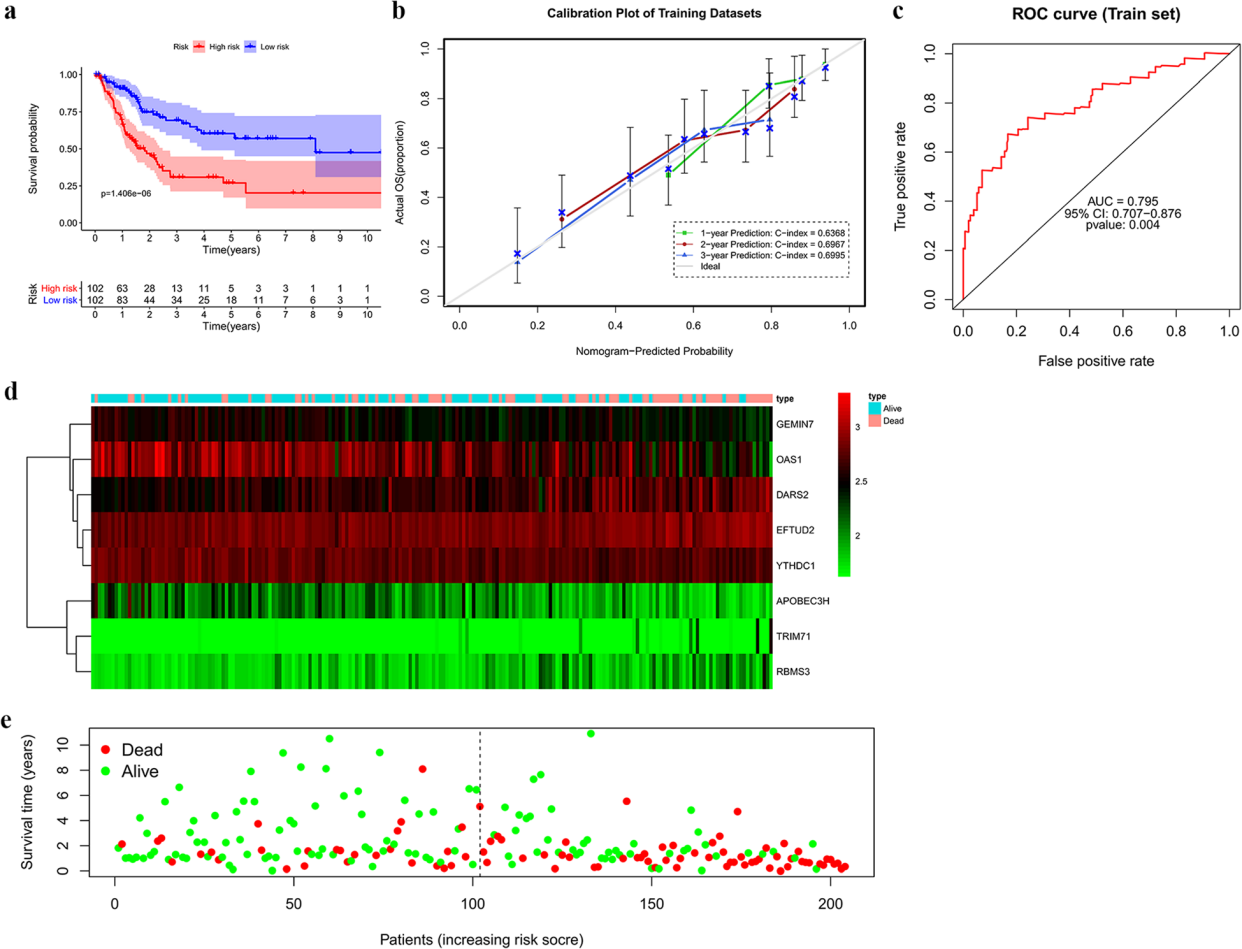
Risk score analysis based on the eight hub RBPs in the TCGA training dataset. **a** Survival analysis of the low- and high-risk subgroups. **b** Calibration curve of the nomogram to predict the probability of overall survival at 1, 2, and 3 years for BLCA patients in the TCGA training dataset. The x- and y-axes represent the predicted and actual overall survival, respectively. **c** ROC curve assessment of the prognostic ability of the risk score model. **d** Heatmap of the expression profiles of the eight hub RBPSs. **e** Survival statuses of BLCA patients with different risk scores

**Fig. 9:**
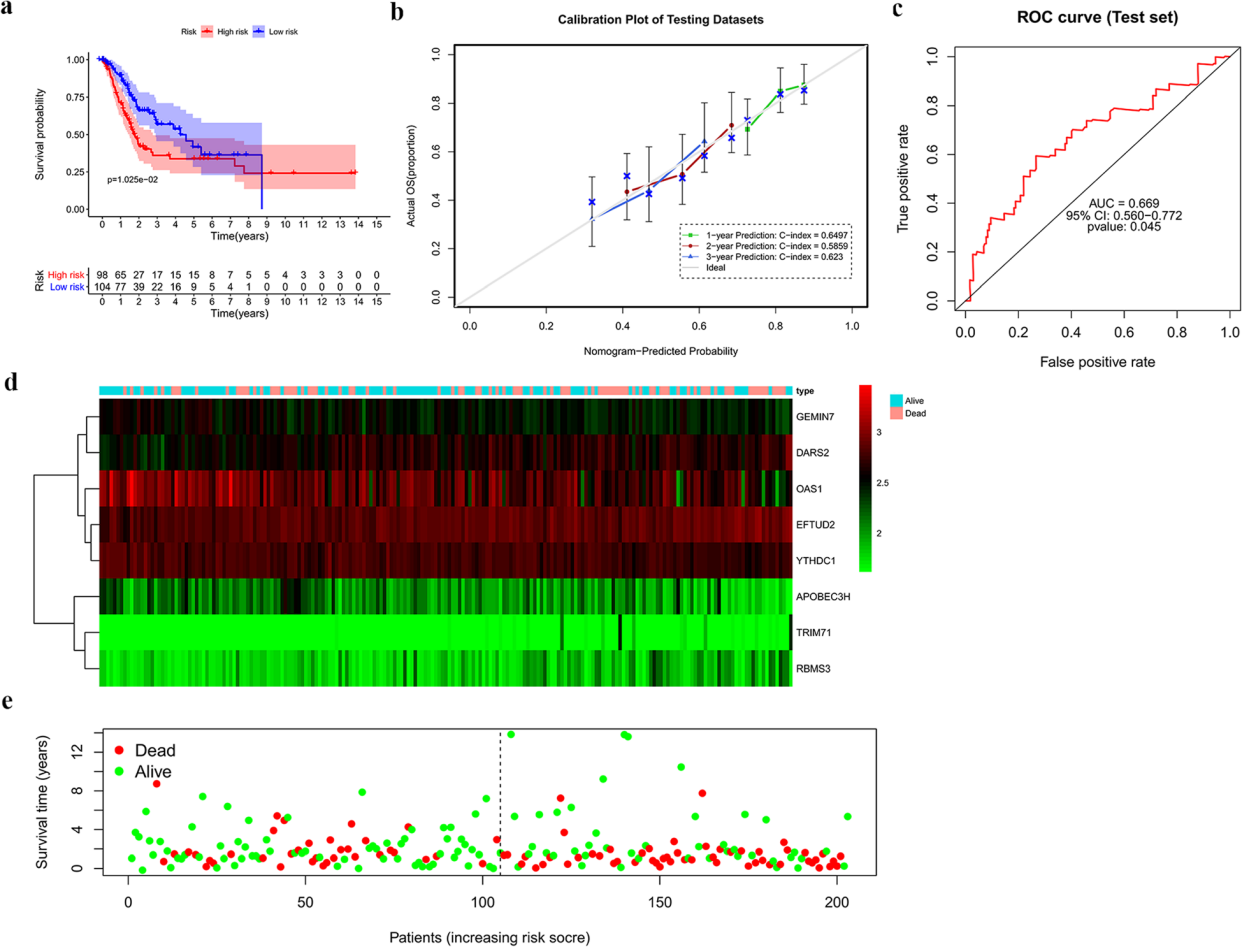
Risk score analysis based on the eight hub RBPs in the TCGA test dataset. **a** Survival analysis of the low- and high-risk subgroups. **b** The calibration curve of the nomogram to predict the probability of overall survival at 1, 2, and 3 years for BLCA patients in the TCGA test dataset. The x- and y-axes represent the predicted and actual overall survival, respectively. **c** ROC curve assessment of the prognostic ability of the risk score model. **d** Heatmap of the expression profiles of the eight hub RBPSs. **e** Survival statuses of BLCA patients with different risk scores

We then applied survival analysis to assess the predictive performance of this model. A total of 407 BLCA patients from TCGA were divided into the training and test datasets containing 204 cases and 203 cases, respectively. We then divided the 204 BLCA patients in the training dataset into low- and high-risk groups according to the median risk score. The result showed that patients in the high-risk group had a poor OS compared with those in the low-risk group (Fig. 8a). In addition, we constructed calibration plots and calculated the C-index, which was respectively 0.6368, 0.6967 and 0.6995 for OS prediction at the 1-, 2-and 3-year, suggesting a good conformity between the predicted and observed outcomes (Fig. 8b). Furthermore, a time-dependent ROC analysis [13], performed to further assess the prognostic power of the risk score model, showed an area under the ROC curve (AUC) of 0.795 (95% confidence interval, 0.707–0.876) (Fig. 8c), indicating the favourable predictive discrimination of the prognostic model. Subsequently, BLCA patients in the training dataset were ranked by risk score to analyse their survival distribution. The heatmap showed the expression profile of the hub RBPs with increasing numbers of dead patients (Fig. 8d). The scatter plot showed that the mortality rate of the patients increased with increasing risk score (Fig. 8e). We also validated the prognostic ability of this model in the test dataset, observing consistent results between the test and training datasets (Fig. 9a-e). These results showed acceptable sensitivity and specificity of the prognostic model.

### Construction of a nomogram

**Fig. 10:**
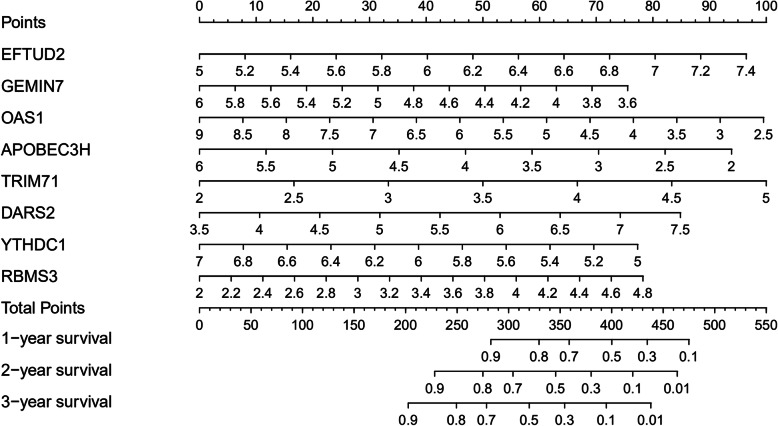
Nomogram predicting 1 -, 2 -, and 3-year OS of BLCA patients in the TCGA

To establish a quantitative prediction method for evaluating BLCA prognosis, we constructed a nomogram based on the eight independent prognosis-associated RBPs (Fig. 10). The point scale in the nomogram was used to assign points to each variable. We drew a vertical line to determine the points for each variable and summed the points of all variables to calculate the total points for each patient, which was then normalized to a distribution of 0 to 100. Hence, we could estimate the survival rates of BLCA patients at 1, 2, and 3 years by calculating the total points for each patient by drawing a vertical line between the total point axis and each prognosis axis. This approach may help clinicians to make clinical decisions for BLCA patients.

### Assessment of the prognostic value of clinical parameters

**Fig. 11:**
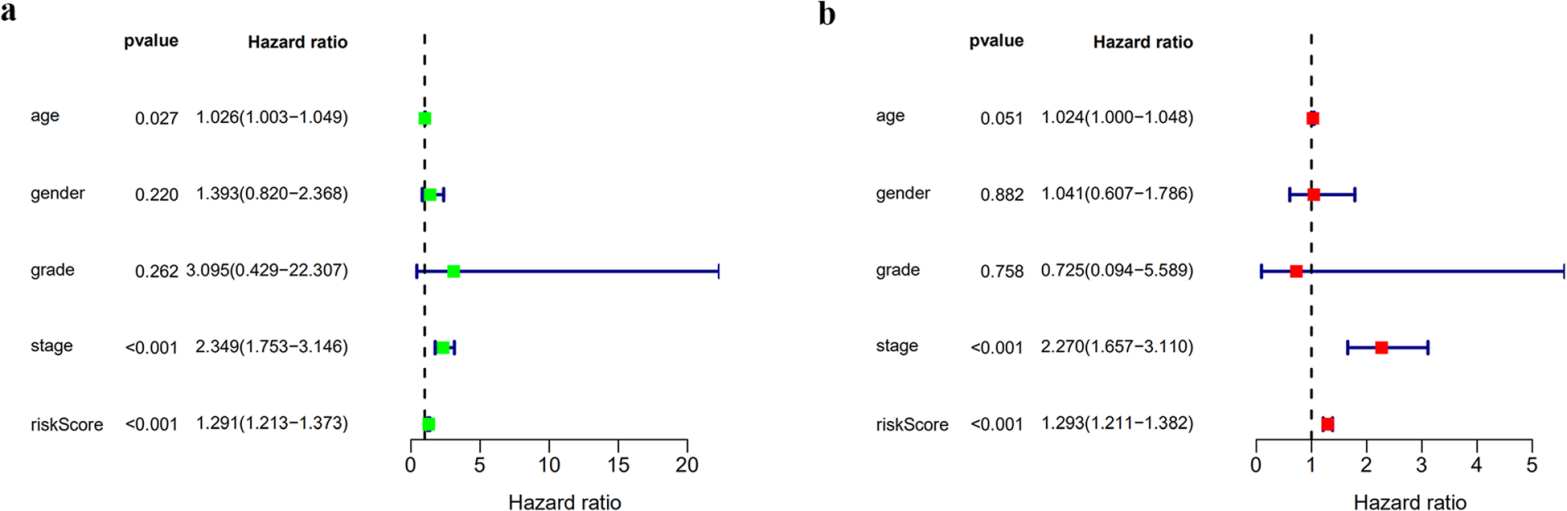
Prognostic value of different clinical parameters. **a** Forest plots of univariable Cox regression analysis. **b** Forest plots of multivariable Cox regression analysis. *P* < 0.05 indicated statistical significance

To further assess the prognostic value of different clinical characteristics in BLCA patients from the TCGA database, we performed Cox regression analysis. The result of univariable analysis showed that age, tumour stage, and risk score were related to OS in BLCA patients (Fig. 11a). In the multivariable Cox regression analysis, only tumour stage and risk score remained independent prognostic indicators for BLCA patients (Fig. 11b).

## Discussion

Increasing evidence has confirmed the role of RBPs in carcinogenesis and some studies have consistently emphasized the association of RBPs as candidate biomarkers for patient prognosis and response to therapy in different cancer types [14–17]. However, how to apply these findings to clinical practice warrants further study. In the present study, we first screened 385 RBPs differentially expressed between BLCA and normal tissues from the TCGA database. Then, we systematically analysed the biological pathways and constructed PPI networks for these differential RBPs. Subsequently, we performed univariable and multivariable Cox regression analyses to further identify eight independent prognosis-associated RBPs. To further understand their biological functions and clinical significance, we also conducted survival and ROC analyses of the eight hub RBPs. Finally, we constructed a risk model based on these eight prognostic hub RBPs to predict the prognosis of BLCA patients. The results of our study provide new biomarkers for prognostic assessment of BLCA patients.

GO enrichment analysis showed that the biological processes (BPs) of the differently expressed RBPs were mainly enriched for ncRNA processing, tRNA metabolic processes, RNA splicing, regulation of mRNA metabolic processes, ribosome biogenesis, and translational regulation. Calo et al. reported that DExD-box helicase 21 (DDX21), a member of the DEAD-box RNA helicase family, was required for pre-rRNA processing; occupied the transcribed rDNA locus; directly contacted both rRNA and snoRNAs; and promoted rRNA transcription, processing, and modification in the nucleolus [18]. Protein quaking (QKI), a splicing factor frequently downregulated in lung cancer and correlated with poor prognosis, selectively suppressed the inclusion of NUMB mRNA exon 12 to promote the expression of a NUMB isoform, thereby inhibiting proliferation and the Notch signalling pathway [19]. Another study reported that DEAH-box helicase 37 (DHX37), an ATP-dependent RNA helicase, was essential for ribosome biogenesis by facilitating small ribosomal subunit maturation. The cellular component analysis in the present study showed that the differential RBPs were primarily enriched for cytoplasmic ribonucleoprotein granule, ribonucleoprotein granule, mitochondrial ribosome, and P-body. Recently, Rozanska et al. demonstrated that ribosome binding factor A (RBFA) was a mitochondrial RBP that played important roles in mitoribosome biogenesis. RBFA combined with helices 44 and 45 of the 12S rRNA in the mitoribosomal small subunit promoted the dimethylation of two highly conserved consecutive adenines, necessary for completing mitochondrial rRNA maturation and promoting the formation of a functional mitoribosome [20]. P-body, a cytoplasmic ribonucleoprotein granule, reportedly played a crucial role in translational repression and mRNA decay [21]. NBDY (NoBody), a recently identified P-body protein, inhibited mRNA turnover, as the silencing of NBDY expression destabilized a reporter of nonsense-mediated decay [22]. YTH N6-methyladenosine RNA binding protein 2 (YTHDF2), another recently discovered P-body protein, is specifically bound to N-methyladenosine and promoted the destabilization of mRNAs with this modification [23]. Regarding molecular function, the differential RBPs in the present study were largely enriched for catalytic activity, acting on RNA, ribonuclease activity, translation factor activity, and RNA and ribonucleoprotein complex binding. For example, mex-3 RNA binding family member C (MEX-3C) is a MEX-3-homologous protein with E3 ubiquitin ligase activity mediated by a RING domain and critical for RNA degradation [24]. Cano et al. demonstrated that MEX-3C regulated *HLA-A2* expression by binding to its 3′-UTR, thereby inducing the ubiquitin-dependent degradation of this mRNA [25, 26].

The KEGG pathway analysis revealed that the differentially expressed RBPs were significantly enriched for aminoacyl-tRNA biosynthesis, methionine metabolism, lysine degradation, and 2-oxocarboxylic acid metabolism. A previous study reported that the faithful translation of genetic information from mRNA to protein is determined by two factors: the availability of aminoacyl-tRNAs composed of cognate amino acid-tRNAs pairs and the accurate selection of aminoacyl-tRNAs on the ribosome. Therefore, aminoacyl-tRNA biosynthesis, which is mediated by aminoacyl-tRNA synthetases, is crucial for translational quality control [27]. However, the role of RBP-mediated aminoacyl-tRNA biosynthesis in BLCA is unclear and warrants further study. AtGRP7, a known circadian clock regulated glycine-rich RBP, is an alternative splicing regulator [28]. Steffen et al. demonstrated that AtGRP7 loss-of-function mutants increased dimethylated lysine 4 levels in histone H3, which are markers of active transcription [29].

Subsequently, we established a PPI network of these differentially expressed RBPs and constructed a module containing 104 key RBPs. Most of these key RBPs have been reported to play important roles in cancer initiation, development, and metastasis. Cancer susceptibility candidate 3 (CASC3), also known as metastatic lymph node 51 (MLN51), is a splicing factor that regulates long intron-containing genes splicing. CASC3 overexpression significantly promoted hepatocellular carcinoma cell proliferation [30]. EFTUD2, an alternative splicing factor, may regulate the innate immune response in macrophages. Silencing of EFTUD2 expression significantly inhibited chronic intestinal inflammation and tumorigenesis, which was related to the reduced production of inflammatory cytokines and tumorigenic factors [31]. DDX39, a DEAD-box RNA helicase, was upregulated in hepatocellular carcinoma tissues and cells and negatively correlated with patient OS. Furthermore, DDX39 overexpression promoted hepatocellular carcinoma cell proliferation and invasion through the Wnt/β-catenin pathway [32]. Block of proliferation 1 (BOP1), which reportedly participates in 28S and 5.8S ribosomal RNA processing and 60S ribosome biogenesis, was down-regulated in patient-derived melanoma samples [33, 34]. A loss of BOP1 also resulted in acquired resistance to BRAF kinase inhibitors in melanoma by increasing MAPK signalling [34]. Importin 4 (IPO4) belongs to the importin β family, which is responsible for transporting histones H3 and H4 into the nucleus for chromatin assembly [35]. Xu et al. reported IPO4 overexpression in gastric cancer tissues and cell lines and demonstrated that IPO4 knockdown suppressed gastric cancer cell proliferation and migration [36]. This brief overview highlights the role of RBPs in tumorigenesis and development. Thus, the regulation of RBPs may represent an important breakthrough in tumour diagnosis, therapy, and prognostic prediction.

In this study, we finally identified eight independent prognosis-associated RBPs and used them to construct a prognostic prediction model. Moreover, the prognostic ability of this model was validated in the test dataset, which also showed a good predictive performance. Although five of the eight hub RBPs did not have statistically significant differences in the multivariate Cox regression analysis, these hub genes were found to be associated with OS in BLCA patients through survival analyses, which means these genes can be used to predict the prognosis of BLCA patients. Therefore, these five hub genes were also included when we constructed prognostic model. Among these hub RBPs, high expression levels of GEMIN7, OAS1, APOBEC3H, and YTHDC1 were associated with favourable prognosis in BLCA patients, while high expression levels of DARS2 and RBMS3 predicted poor prognosis. A previous study reported that GEMIN7, a component of the survival motor neuron complex, is involved in the biogenesis of the small nuclear ribonucleoprotein complex [37]; however, its role in cancers is rarely reported. OAS1, initially identified as an interferon-induced antiviral enzyme, was recently associated with 5-azacytidine (AZA) sensitivity, the deficiency of which resulted in the NCI-60 set of cancer cell lines resistant to AZA [38]. APOBEC3H is a single-stranded DNA cytosine deaminase that can induce mutations in tumour cells, resulting in immune recognition or cancer cell death [39]. YTHDC1, a N^6^-methyladenosine binding protein localized in YT-bodies adjacent to nuclear speckles, regulates mRNA splicing by recruiting splicing factors to the targeted mRNA [40]. DARS2 promoted cell cycle progression and inhibited hepatocellular carcinoma cell apoptosis via the miR-30e-5p/MAPK/NFAT5 pathway [41]. Wu et al. demonstrated that the loss of RBMS3 in epithelial ovarian cancer not only induced chemoresistance to platinum but also promoted recurrence via miR-126-5p/β-catenin/CBP signalling. Moreover, the loss of RBMS3 was associated with poor overall and relapse-free survival in epithelial ovarian cancer patients [42]. Another study found that RBMS3 inhibited breast cancer cell proliferation, migration, and invasion through the Wnt/β-catenin signalling pathway [43]. The loss of EFTUD2 repressed colonic inflammation and tumorigenesis via inactivation of NF-κB signalling [31]. TRIM71, an E3-ubiquitin ligase, induced ubiquitination and degradation of mutant p53 by binding to its transactivation domain in ovarian cancer, leading to decreased ovarian cancer cell growth [44]. However, the functions and molecular mechanisms of these hub RBPs in BLCA remain poorly understood; thus, functional experiments are needed to further explore their potential roles and mechanisms. Moreover, large sample and multi-centre clinical studies are expected to verify the results.

In summary, our study systematically analysed the expression and prognostic value of differentially expressed RBPs in BLCA using a series of bioinformatics techniques. We finally identified eight independent prognosis-associated RSPs and successfully constructed a prognostic risk score model to effectively assess the prognosis of BLCA patients. To our knowledge, this is the first study to develop an RBP-related prognostic model for BLCA. This study provides a basis for the development of new therapeutic targets and prognostic biomarkers.

## Conclusion

We developed a prognostic model for BLCA patients and validated the performance of the model, which might facilitate the development of new biomarkers for the prognostic assessment of BLCA patients.

## Data Availability

The datasets used and/or analysed during the current study are available from the corresponding author on reasonable request. No additional permissions were required to use any of the repository data.

## Abbreviations

APOBEC3H: Apolipoprotein B mRNA editing enzyme catalytic subunit 3H
AUC: Area under the ROC curve
AZA: 5-azacytidine
BLCA: Bladder cancer
BOP1: Block of proliferation 1
BPs: Biological processes
CASC3: Cancer susceptibility candidate 3
CT: Computed tomography
DARS2: Aspartyl-tRNA synthetase 2, mitochondrial
DDX21: DExD-box helicase 21
DHX37: DEAH-box helicase 37
FC: Fold-change
FDR: False discovery rate
GEMIN7: Gem nuclear organelle associated protein 7
GO: Gene ontology
HPA: Human Protein Atlas
IMP: Insulin-like growth factor II messenger RNA binding protein
IPO4: Importin 4
KEGG: Kyoto Encyclopedia of Genes and Genomes
MEX-3C: Mex-3 RNA binding family member C
MCODE: Molecular Complex Detection
MLN51: Metastatic lymph node 51
NBDY: NoBody
OAS1: 2′-5′-oligoadenylate synthetase 1
OS: Overall survival
PPI: Protein-protein interaction
QK1: Protein quaking
RBFA: Ribosome binding factor A
RBMS3: RNA-binding motif, single-stranded-interacting protein 3
RBPs: RNA-binding proteins
ROC: Receiver operator characteristic
TCGA: The Cancer Genome Atlas
TRIM71: Tripartite motif containing 71
YTHDC1: YTH domain containing 1
YTHDF2: YTH N6-methyladenosine RNA binding protein 2
